# Thermodynamically optimal whole-genome tiling microarray design and validation

**DOI:** 10.1186/s13104-016-2113-4

**Published:** 2016-06-13

**Authors:** Hyejin Cho, Hui-Hsien Chou

**Affiliations:** Department of Genetics, Development and Cell Biology, Iowa State University, Ames, IA 50011 USA; Department of Computer Science, Iowa State University, Ames, IA 50011 USA

**Keywords:** Tiling microarray design, Prokaryote transcriptome, Thermodynamics, Hybridization, PICKY software, Microarray validation

## Abstract

**Background:**

Microarray is an efficient apparatus to interrogate the whole transcriptome of species. Microarray can be designed according to annotated gene sets, but the resulted microarrays cannot be used to identify novel transcripts and this design method is not applicable to unannotated species. Alternatively, a whole-genome tiling microarray can be designed using only genomic sequences without gene annotations, and it can be used to detect novel RNA transcripts as well as known genes. The difficulty with tiling microarray design lies in the tradeoff between probe-specificity and coverage of the genome. Sequence comparison methods based on BLAST or similar software are commonly employed in microarray design, but they cannot precisely determine the subtle thermodynamic competition between probe targets and partially matched probe nontargets during hybridizations.

**Findings:**

Using the whole-genome thermodynamic analysis software PICKY to design tiling microarrays, we can achieve maximum whole-genome coverage allowable under the thermodynamic constraints of each target genome. The resulted tiling microarrays are thermodynamically optimal in the sense that all selected probes share the same melting temperature separation range between their targets and closest nontargets, and no additional probes can be added without violating the specificity of the microarray to the target genome.

**Conclusions:**

This new design method was used to create two whole-genome tiling microarrays for *Escherichia coli* MG1655 and *Agrobacterium tumefaciens* C58 and the experiment results validated the design.

**Electronic supplementary material:**

The online version of this article (doi:10.1186/s13104-016-2113-4) contains supplementary material, which is available to authorized users.

## Findings

### Background

Different types of microarray exist, and they all have DNA probes on the microarray surface to hybridize, or capture, targeted sequences in the samples that are poured over them. Microarrays differ on how their probes are designed and what are their intended targets. The most common microarrays are designed to detect gene expressions; their probes are designed according to annotated gene sets and are used to detect individual gene expressions. Although gene expression microarrays have been in use for more than a decade and have produced a large volume of biological data, they are gradually being replaced by next-generation sequencing (NGS) techniques because NGS techniques can detect novel RNA transcripts and provide a better dynamic range of measured gene expression values [1].

Nevertheless, microarrays are still being used in some other applications. For example, sequence fragment capturing microarrays work by grabbing specific genome fragments or RNA transcripts of interest to researchers, hence enriching the targeted samples; the captured fragments can then be sequenced and analyzed using NGS techniques [2, 3]. In this work, we focus on another application where microarrays are still viable—the whole-genome tiling microarrays. A tiling microarray is designed against a genome, not a gene set, and can be used to detect all transcription activities from either annotated genes or novel transcripts; the latter may include short regulatory RNAs that are the interest of recent studies [4–6].

We have developed a new tiling microarray design method based on the whole-genome thermodynamic analysis software Picky that was previously developed to design traditional microarrays [7–9]. We then used our new method to design two whole-genome tiling microarrays for *Escherichia coli* (*E. coli*) MG1655 and *Agrobacterium tumefaciens* (*A. tumefaciens*) C58. Picky can analyze a whole genome to identify thermodynamically unique probes. The melting temperatures of each probe candidate with its intended target and with its closest nontargets anywhere in the genome are calculated by Picky according to the thermodynamic nearest-neighbor models of prefect matches [10], mismatches [11–14], bulges [15], and dangling-ends [16]. The equations used by Picky are deterministic according to thermodynamic principles, and Picky exhaustively applies these equations to all potential probe-target as well as probe-nontarget hybridizations. Picky design qualities have been quantitatively validated [17].

The main advantage of Picky over sequence-level comparison software such as BLAST [18] is its precision. Although BLAST is often used to estimate probe specificity by calculating its identity and match length to nontargets [19, 20], these estimates are less precise than thermodynamics. For example, we screened a previous tiling microarray probe set containing 409,807 probes for a bacterial species using Picky [21], and found 21,773 (5.3 %) of its probes have the potential to hybridize to nontargets (output W83.picky in Additional file 1). In Table 1, sample data from this probe set shows that probes of the same length (50 bp), the same identity to nontarget [24 bp (base-pair); <50 %] and the same match stretch to nontarget (14 bp; <30 %) can still have a wide estimated melting temperatures range from 28 to 68 °C. Furthermore, the lowest probe-to-target melting temperature at 54.33 °C is much lower than the highest probe-to-nontarget melting temperature at 68.56 °C. Essentially, it is impossible to set a single microarray hybridization temperature that allows all probes to function effectively. This probe set was designed using a sophisticated pipeline involving BLAST to screen for cross-hybridizations and was considered optimal by that standard [22]. Nevertheless, thermodynamic equations are inherently nonlinear, thus a wide range of melting temperatures can still be calculated from the same sequence-level identities.

**Table 1:** Sequence-level comparisons cannot faithfully predict thermodynamic properties

Probe (top strand) and nontarget (lower strand) match (complementary bases in uppercase; mismatched bases in lowercase)	Nontarget match identity (bp)	Nontarget match stretch (bp)	Probe to nontarget melting temp. (°C)	Probe to target melting temp. (°C)
tagagtagAAaaaCAAataAaAGAcattaaAGAAAATGATTTTTgattTttgtgttagTTaccGTTacgTgTCTcacgccTCTTTTACTAAAAAaagtAt	24	14	28.39	55.14
CTtgAaaTtgaaTacAaattctaTaaaTCAATGATATGAATacaataACAGAtgTggAtagaAgcTacctcgaAgggAGTTACTATACTTActcaccTGT	24	14	29.92	54.33
TcaAAgtctAtgatAttcgacAtAtaaTctTGAATCGAAAAAACaGCctcAagTTtctcTtaccTgtctgaTgTtgaAatACTTAGCTTTTTTGaCGcct	24	14	38.04	61.27
cGGTGCTCGATACGAttGCcCtgatgcTGacaaggCttctaTcgAaTCtcaCCACGAGCTATGCTctCGaGggccacACaagcggGttccgAagTaAGga	24	14	48.01	72.36
agcagTcgCtAcCGcttgcCGGACGAATTGCCGgTCGctccTgtttGggcctaaaAaaGcTcGCattttGCCTGCTTAACGGCaAGCacaaAaggcCttt	24	14	58.02	80.75
AacGgAGAaggaGagTgCcgggcGGAAGCCGGCGGCGaaaaCgTccaccgTgcCgTCTaggaCatAgGcttacCCTTCGGCCGCCGCacggGtActgcta	24	14	64.30	82.65
gGCCGGTGGCGGCCGtGagatgtcgctctCggcGAatgGCatTctgAtCTaCGGCCACCGCCGGCtCaaatgttatctcGgaaCTacaCGctAcggTgGA	24	14	68.56	80.10

Our goal in this work is to adapt Picky, which was originally developed to take annotated gene sets as input and design traditional microarrays, for the design of tiling microarrays. We hope to achieve maximum probe coverage of the genome while maintaining the same thermodynamic specificity of Picky designed probes. After the tiling microarrays for *E. coli* MG1655 and *A. tumefaciens* C58 were designed using our new method and manufactured, the two bacteria were grown under 10 different treatment conditions to trigger gene expression changes. Subsequently, samples extracted from them were applied to the two tiling microarrays to validate their design quality and also to uncover novel transcripts.

## Methods

### Sample procurement and genome confirmation

The *E. coli* MG1655 strain was obtained from CGSC *E. coli* genetics resources at Yale University (CGSC #6300) [23]. The *A. tumefaciens* C58 strain was obtained from Dr. Kan Wang’s lab at Iowa State University [24]. Bacteria were recovered from the delivery medium and grown under standard conditions (37 °C in Luria–Bertani medium for MG1655 and 28 °C in YEP medium for C58). The QIAGEN DNeasy blood & Tissue kit (#69504) was used to extract total DNA from both bacteria. The Qubit 2.0 Flurometer was used to precisely quantify DNA concentration in the samples and the Experion DNA 12 K Analysis Kit was used to check the DNA quality. The total DNA was eluted in 100 uL buffer and 50 uL of that was sent for sequencing confirmation.

The genomes of the two bacteria MG1655 and C58 were resequenced using the Illumina HiSeq 2000 instrument and de novo assembled using the Velvet software [25]. Minimus2, which is part of the AMOS software package, was used to merge Velvet contigs to form longer scaffolds [26]. BLAT was then used to align merged contigs to the reference genomes [27]. The alignment is important to correctly orient some contigs, find repeated contigs and fill in the gaps among aligned contigs. The reference genomes were used to guide the assembly of the contigs, but not the individual reads. The AT plasmid of C58 was not successfully assembled due to lack of matched contigs, thus the reference sequence was used in subsequent design.

We have found hundreds of single nucleotide polymorphisms between the assembled genomes and the reference genomes, which support our initial concern that the bacteria we obtained might not match the reference genome sequences exactly. These polymorphisms, which are summarized in Table 2, might cause slightly less precise tiling microarray design if left unidentified. The resequencing confirmation step is entirely optional but it helps improve the tiling microarray design quality. The resequencing data can be obtained from NCBI short read archive database with accession numbers SRX806374 and SRX806654 and the assembled new genomic sequences are provided in Additional file 2.

**Table 2:** Polymorphisms between lab bacteria genomes and official GenBank reference genomes

Bacteria	Genome size (bp)	Single nucleotide polymorphisms	Identity to GenBank reference genome (%)
*E. coli* MG1655	4,639,675	191	100.00
*A. tumefaciens* C58	5,746,078	203	100.00

### Tiling microarray design

Based on the assembled genome sequences, we designed the two whole-genome tiling microarrays for *E. coli* MG1655 and *A. tumefaciens* C58 using PICKY [9, 28]. The design process is summarized as follows. The genome sequences were broken up into 100 bp fragments without overlaps—these were treated as gene targets for probe design to ensure even distribution of the tiling probes. Separately, 50 bp fragments centered on the boundaries between the target fragments (25 bp on either side of a boundary) were extracted and treated as unintended targets for probe design (i.e., fragments to avoid) to ensure that tiling microarray probes will not inadvertently target the boundaries between fragments. PICKY was run using both the targets and unintended fragments as input. The benefit of this approach is that we can take full advantage of the probe specificity calculation offered by PICKY while making it design tiling microarrays with evenly distributed probes. The following parameters specific to tiling microarray design were given to PICKY: maximum match length 18, minimum match length 8, minimum sequence similarity 66 %, and minimum melting temperature difference 5 °C. Any probe candidate with the maximum match to any off-targets are automatically ruled out for further consideration. Probe candidates with the minimum match to any off-targets are thermodynamically screened by extending around the matched region up to the minimum sequence similarity level to estimate its melting temperature with potential cross-hybridization off-targets; probe candidates with less than the minimum melting temperature difference between its target and its closest off-target will not be selected. All other PICKY parameters were taken at their default values, including the screening of both strands of each input sequence to ensure probe specificity in either direction. The minimum and maximum match length parameters and the minimum sequence similarity parameter ensure that a wide range of nontarget matches will be screened thermodynamically by PICKY. The minimum melting temperature difference ensures that only probes unique to the target fragments will be selected.

After running PICKY the first time, it turned out that some target fragments did not have matching probes under the stringent design parameters. To increase the number of useful probes, we ran PICKY again with the following new input. The target fragments were separated into two different sets: one containing fragments without probes as a new target set, and the other containing fragments that had probes designed for them during the first PICKY run. The second set was combined with a modified boundary fragment set to form the new unintended fragment set. The modified boundary fragments were shortened to 40 bp centered on the boundaries between the target fragments. The second PICKY run used the same parameters as in the first run, but it produced additional probes because the shortened boundary fragments allow more borderline probes to be selected.

### Microarray manufacturing

Microarray probes obtained from both PICKY runs were merged to obtain the final design output. When designing tiling microarray for the C58 bacterium, the pTi plasmid of C58 was also added to the design data set to increase the versatility of the C58 tiling microarray. We have chosen the NimbleGen Custom Microarray Service to manufacture the tiling microarrays. The NimbleGen microarray platform has a synthesis cycle limitation of 148 on custom designed microarray probes [29]. Therefore, 19 MG1655 and 20 C58 probes were removed because they exceeded the limit.

Probes for the exogenous gene hygromycin to *E. coli* and *A. tumefaciens* were added: 1000 hygromycin probes were added to the C58 probe set and 2000 hygromycin probes were added to the MG1655 probe set. These probes can be used as quality controls if the *hygromycin* gene is added to each bacteria sample during the microarray hybridization protocol to help detect any technical bias. The manufacturer also added other control probes to the final probe set for proprietary quality control and microarray image alignment. All probes are synthesized in situ on the NimbleGen microarray surface using the 4 × 72 K microarray layout, meaning that there are four independent microarrays per each NimbleGen glass chip and each microarray contains up to 72 K probes. Final results of the microarray design are summarized in Table 3. Complete microarray design information and experiment data were deposited into the NCBI gene expression omnibus (GEO) database with the Series Access Number GSE61738, which is a super series combining both microarray series for MG1655 and C58.

**Table 3:** Results of tiling microarray design

	MG1655	C58
Microarray unique probe count	67,435	71,498
Avg. probe length (bp)	41	40
% of 100-bp fragments without useful probes	6.13	4.48
% of genome covered by probes	93.87	95.52
Hygromycin control probes	2000	1000

Although NimbleGen has exited the custom microarray manufacturing business, the tiling microarrays can still be manufactured by other manufacturers given the original microarray design information. Naturally, some array-specific protocols such as labeling and image quantification may need to be modified accordingly if different microarray platforms are used.

## Results and discussion

### Microarray validations

#### Experiment protocol

*E. coli* MG1655 and *A. tumefaciens* C58 cells were grown under 10 different treatments listed in Table 4 (1 standard and 9 stressed conditions). The significantly varied growth conditions help induce large-scale gene expression changes that ideally should cover most of the transcriptome landscape of the two species. Cells were harvested after treatment at the harvest point given in Table 4.

**Table 4:** Growth conditions of MG1655 and C58

Name	Conditions	Harvest point
*E. coli* MG1655		
Standard	Grown at 37 °C in LB media	Reached mid-log phase O.D. 600 nm 0.6 ~ 0.8
Cold shock	Grown at 15 °C for 4 h then grown at 37 °C in LB media	Reached half of O.D. 600 nm of Standard
Heat shock	Grown at 50 °C for 4 h then grown at 37 °C in LB media	Same as above
Low pH	Grown at 37 °C for 1 h in LB media with pH4.5 then grown at 37 °C in LB media	Same as above
UV treat	Exposed to UV light for 15 min then grown at 37 °C in LB media	Same as above
Low carbon	Grown at 37 °C in minimal C source MOPS media [49] (C- MOPS)	Same as above
Low nitrogen	Grown at 37 °C in minimal N source MOPS media (N- MOPS)	Same as above
Low C & N	Grown at 37 °C in minimal C and N source MOPS media (C-N- MOPS)	Reached quarter of O.D. 600 nm of Standard
Oxidative	Growth at 37 °C in 49 mL MOPS media with 400 μL 7 % Hydrogen peroxide	Reached half of O.D. 600 nm of Standard
Osmotic	Growth at 37 °C in 45 mL MOPS media with 6 mL 4 M Sodium Chloride	Same as above
*A. tumefaciens* C58		
Standard	Grown at 28 °C in YEM media	Reached mid-log phase O.D. 600 nm 0.6 ~ 0.8
Cold Shock	Grown at 17 °C for 13 h then grown at 28 °C in YEM media	Reached half of O.D. 600 nm of Standard
Heat shock	Grown at 40 °C for 11 h then grown at 28 °C in YEM media	Same as above
Low pH	Grown at 28 °C in AB5.5 media [50]	Reached mid-log phase
Low Iron	Grown at 28 °C in AB7 without Fe media (AB7 Fe-) [51]	Same as above
Oxidative	Grown at 28 °C in 40 mL YEM media with 130 μL 1 % Hydrogen peroxide	Reached half of O.D. 600 nm of Standard
Cold shock & oxidative	Grown at 17 °C for 13 h then grown at 28 °C in YEM media	Reached quarter of O.D. 600 nm of Standard
Low pH & cold shock	Grown at 17 °C for 13 h then grown at 28 °C in AB5.5 media	Same as above
Low pH & heat shock	Grown at 40 °C for 11 h then grown at 28 °C in AB5.5 media	Same as above
Low pH & low Iron	Grown at 28 °C in AB5.5 without Fe media (AB5.5 Fe-)	Same as above

**Fig. 1:**
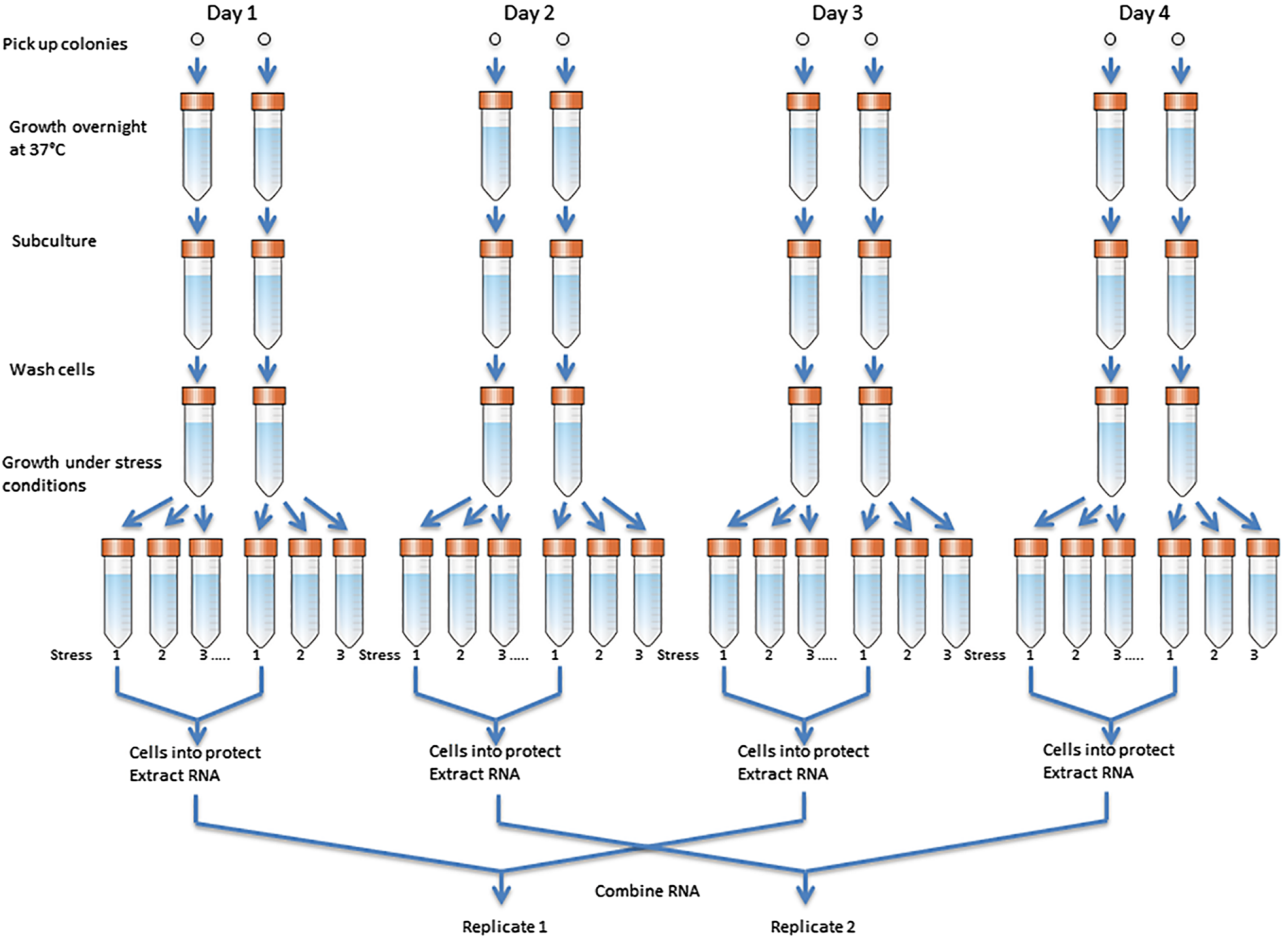
Processing of biological replicates. In each day, two culture tubes under each treatment condition were combined and the RNA sample was extracted from the combined tube. The extracted RNA samples from 2 different days (days 1 and 3, or days 2 and 4) were later combined to become the final replicate

To control noise and bias, samples were pooled, randomized, blocked and replicated. We treated each microarray as a statistical ‘block’ and randomly placed samples onto microarrays to balance variances from batch processes and positional effects [30]. Two biological replicates were produced for each bacterium under each treatment condition. The biological replicates were prepared by pooling samples according Fig. 1; each biological replicate eventually was made from 4 different cell cultures grown at 2 different days [31].

Two biological replicates were performed for each species under each treatment condition. The Qiagen RNeasy Mini Kit (#74104) was used to purify total RNA after on-column DNase digestion to remove DNA contaminations (#79254). The total RNA were revere-transcribed to cDNA using Life Technologies Superscript double stranded cDNA synthesis kit (#11917-020) with a random primer set (#48190-011). Residue RNA were then removed using RNase H (NEB #M0297). The cDNA samples were labeled using the NimbleGen One-Color Labeling Kit (#06370411001) and quantified using a Nanodrop ND-1000 spectrophotometer.

Microarray hybridizations were carried out on a NimblemGen Hybridization Workstation 4 (#05223652001) after dissolving labeled cDNA samples in the Hybridization Kit (#05583683001) with appropriate Sample Tracking Controls added (#05223512001). After the manufacturer recommended overnight hybridization (about 16 h), microarrays were washed with the NimbleGen Wash Buffer Kit (#05584507001) and scanned using a GenePix 4100A Microarray Scanner at the maximum resolution of 5 µm for the Cy3 channel.

Scanned microarray images, which contained 4 microarrays on each chip, were processed using the NimbleGen DEVA 1.2.1 software [32]. The DEVA software aligned and anchored the microarray images using special alignment probes on the microarray surface and then split the images into 4 subarrays for the 4 × 72 K NimbleGen layout before quantifying them into individual probe values. Although manual alignments can be performed, we found it unnecessary for all the microarray images processed. The DEVA software also provided automatic RMA normalizations (robust multi-array analysis) across each set of microarray data for MG1655 and C58 to reduce outliers and make data comparisons more meaningful. Two microarray samples were removed from the final data sets due to large bubbles on the microarray surface. In all, 38 microarray samples (18 for MG1655 and 20 for C58) were used for the following validation analyses. The data can be obtained from NCBI GEO database using the Super Series Access Number GSE61738.

#### Probe level consistency validations

For each strain and treatment condition that has two successful biological replicates, we calculated Spearman’s correlation and concordance correlation coefficient (CCC) between them to validate the consistency of the microarrays under biological replicates. Spearman’s correlation was used to measure the reproducibility of two replicates [33]. Concordance correlation coefficient (CCC) provided a better indicator of the accuracy and precision of agreement between two biological replicates [34]. Biological replicates are more variable than technical replicates because the two biological samples were independently grown, harvested and subjected to microarray protocols, thus correlation between biological replicates can be as low as 30 % [35].

**Fig. 2:**
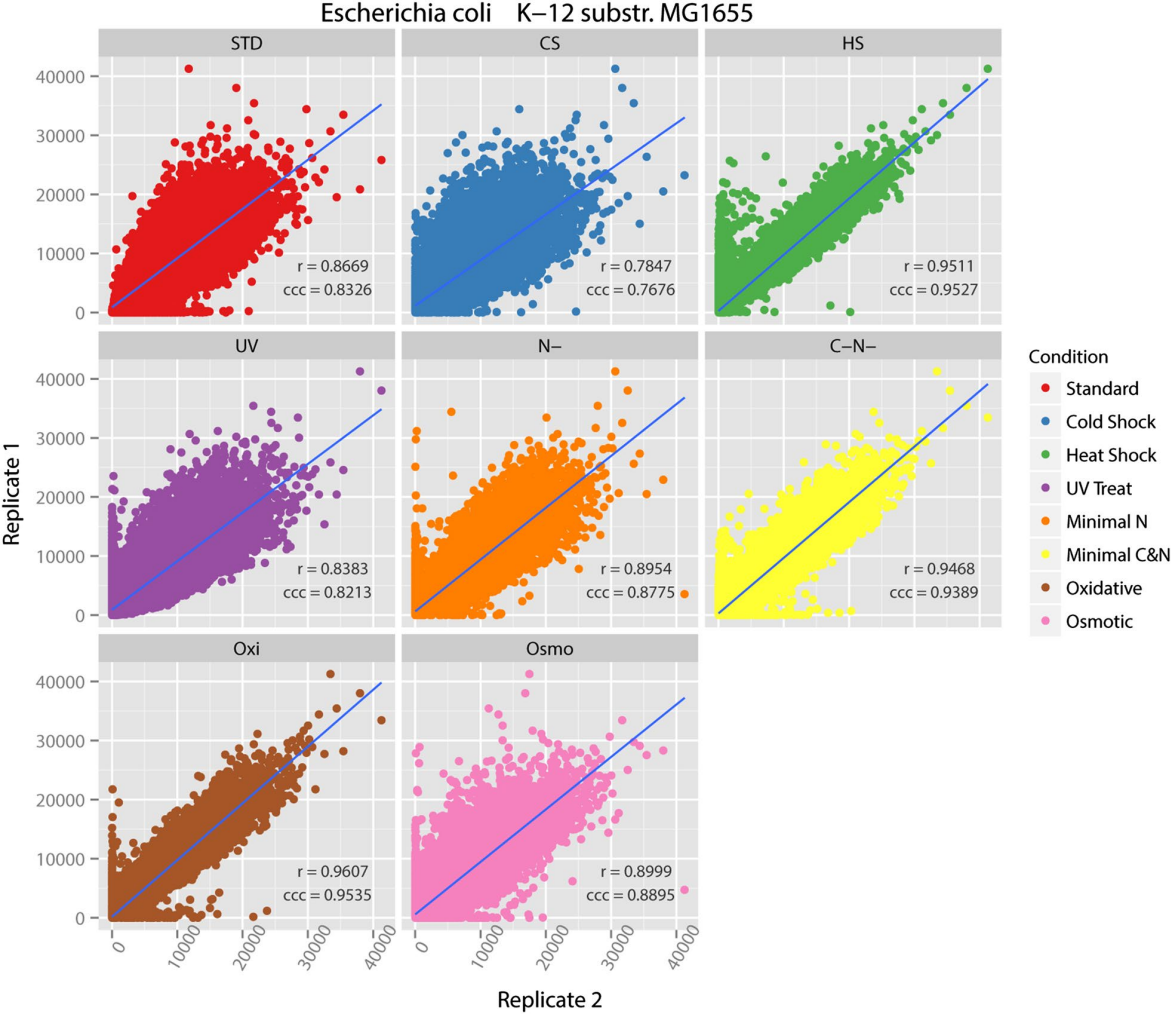
Scatterplots with correlation values for 8 treatment replicate pairs of MG1655. On the graphs, ‘r’ denotes Spearman’s correlation and ‘ccc’ denotes concordance correlation coefficient

Scatterplots for each pair of replicates with regression line and correlation coefficients are shown in Fig. 2 for *E. coli* MG1655. Both statistical tests were performed with 95 % confidential level. The oxidative condition shows the highest correlation values. The cold shock condition has the lowest correlation values, but its Spearman’s correlation value 0.7847 and CCC value 0.7676 still indicate high correlations between the replicates. Therefore, we conclude that the biological replicates of *E. coli* MG1655 under all treatment conditions are consistent enough to indicate that the MG1655 tiling microarray is reliable and reproducible at the individual probe level.

**Fig. 3:**
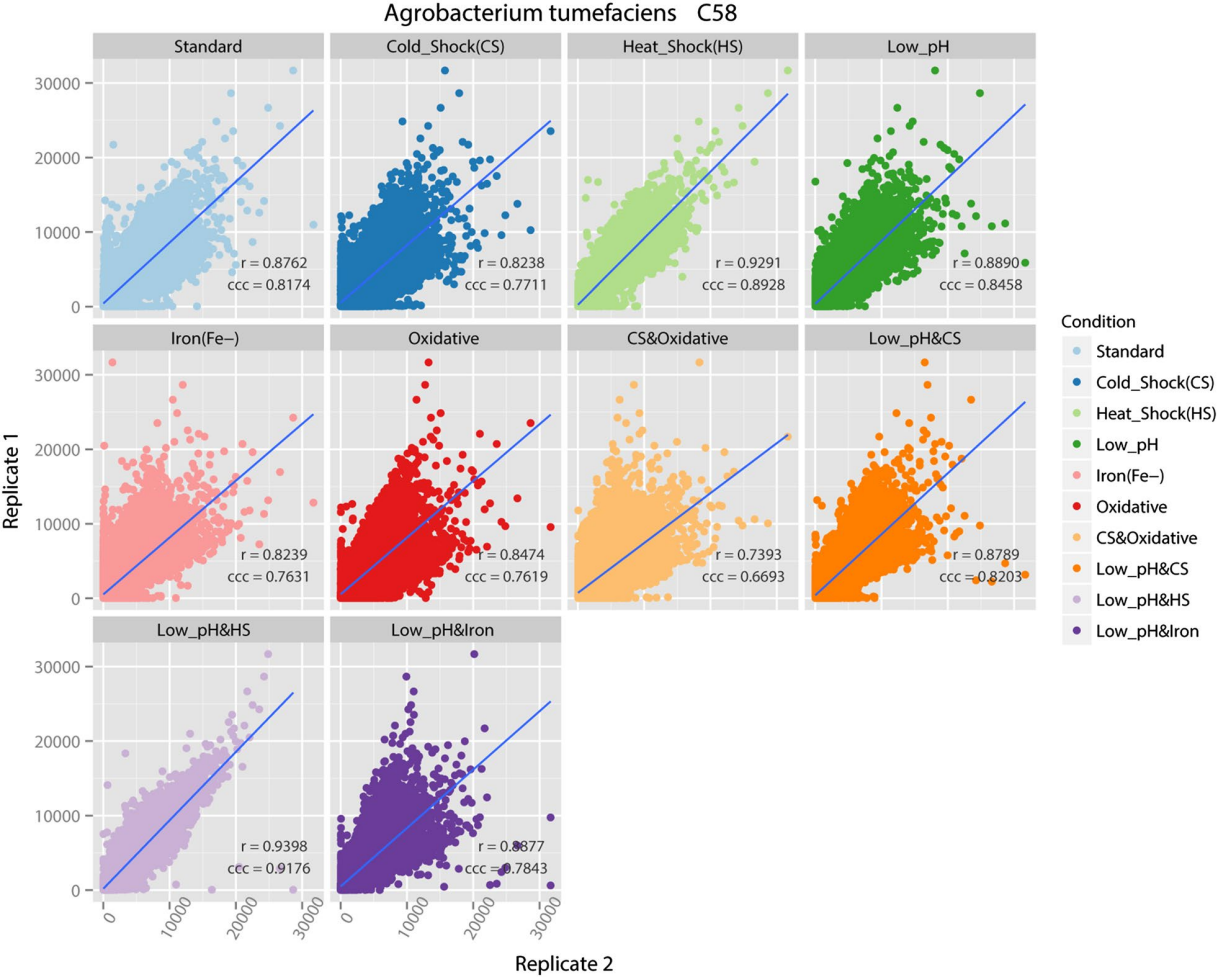
Scatterplots with correlation values for 10 treatment replicate pairs of C58. On the graphs, ‘r’ denotes Spearman’s correlation and ‘ccc’ denotes concordance correlation coefficient

Scatterplots for each pair of biological replicates with regression line and correlation coefficients for *A. tumefaciens* C58 are similarly shown in Fig. 3. They show that the two biological replicates under each treatment condition are consistent with each other with a high correlation value at the 95 % confident level. The low pH and heat shock condition produced the highest correlation value of 0.9398. The cold shock and oxidative condition produced a lower correlation value of 0.7393, which is still high enough to conclude the replicates are highly related. Therefore, we also conclude that the C58 tiling microarray is also reliable at the probe level.

#### Gene level consistency validations

Significant analysis of microarray (SAM) [36, 37] and one-way ANOVA [38] were conducted to detect differentially expressed genes in *E. coli* MG1655 and *A. tumefaciens* C58 under the 10 treatment conditions. As stated earlier, most of the conditions are stress conditions that can promote stress gene responses. The two statistical analyses were performed to validate that the tiling microarrays can detect biologically significant gene expression changes. SAM detects differentially expressed genes across all 10 conditions for each bacterium. One-way ANOVA tests were performed for a few sets of probes targeting some known stress related genes to confirm that their means differ across the 10 conditions for each bacterium, which also identifies the stress responses of cells.

SAM was performed with unpaired two class (control and treatment), delta value of 0.06 and fold change of 2. It found 34 differentially expressed genes in *E. coli* MG1655, including 22 known stress response genes such as *dnaX* [39], *entF* [40], *groL* [41], and *copA* [42, 43]. For *A. tumefaciens* C58, SAM was ran with unpaired two class (control and treatment), delta of 0.065 and fold change of 3 to limit the number of differentially expressed genes reported. It detected 46 differentially expressed genes, including 16 known stress genes such as *livJ* [44], *dadA* [45], and *rpoH* [46]. The one-way ANOVA tests were performed with small subsets of known stress response genes at the 95 % confidential level. Stress response genes detected by SAM and/or confirmed by one-way ANOVA test are summarized in Table 5.

**Table 5:** Detected stress response genes in MG1655 and C58

Gene	Description	Detection
*Escherichia coli* K12 substr. MG1655		
*dnaX*	Temperature sensitive for replication and growth [39]	SAM, ANOVA
*groL*	Acid tolerance response [41]	SAM, ANOVA
*hslO*	Heat shock response [52]	SAM
*entF*	Transcriptional regulation in C and N limited cultures [40]	SAM, ANOVA
*copA*	Essential element in copper homeostasis and copper proteins; involved in oxidative stress protection [42, 43]	SAM
*cusS*	Copper tolerance in anaerobic [43]	SAM
*nusA*	Cold shock response [53]	ANOVA
*uvrA*	DNA repair and SOS response [54]	ANOVA
*aceE*	Induce the oxidative and acid resistance gene yfiD [40]	ANOVA
*kat* ^*f*^	Control of catalase-hydroperoxidase [55]	ANOVA
*cydA*	Control cytochrome bd oxidase on LB but not on minimal medium [56]	ANOVA
*mreBCD*	Involved in cell shapping and osmotolerant [57]	ANOVA
*Agrobacterium tumefaciens C58*		
*cspA*	Cold shock protein [4]	SAM
*rpoH*	Temperature sensitive, control heat shock protein [46]	SAM
*groEL*	Heat shock protein (stress protein) [58]	SAM, ANOVA
*dnaK*	Heat shock protein [59] but also induced by other stresses [58]	SAM, ANOVA
*livJ*	ABC transporter associated with the uptake of metal ions and involved in antioxidative stress defense [44]	ANOVA
*dadA*	Catalyzes the oxidative deamination of D-amino acids [45]	SAM
*fepC*	Outermembrane receptor [60]	ANOVA
*vir* ^a^	Virulence genes induced under several stresses, such as acidic condition or mitomycin C attack [58]	SAM, ANOVA
*chv* ^a^	Induced by acidic pH [50]	ANOVA
*sit* ^a^	Related to iron uptake [61]	ANOVA

^a^Means gene families

After ANOVA tests, multiple pairwise comparison tests (Tukey HSD [38] and Dunnett’s test [37]) were conducted at 95 % confident level as post hoc tests to find out which pairs of treatment conditions have distinctive stress gene expression differences. Differentially expressed stress genes tested and detected for each pair of conditions are listed in Table 6 for *E. coli* MG1655 and Table 7 for *A. tumefaciens* C58. All differentially expressed stress genes confirmed by ANOVA were also found by the post hoc tests except the ‘*fepC*’ gene in *A. tumefaciens* C58. The *p* value of *fepC* from ANOVA is 0.00207 but Tukey HSD or Dunnett’s test cannot identify it as differentially expressed in all pairs of conditions. It may be inferred that the means for this gene in all conditions are different from each other and there is no pair of conditions that is significantly different to allow detection by the post hoc tests.

**Table 6:** Differentially expressed stress genes in each pair of conditions for MG1655

	SD	CS	HS	pH	UV	C-	N-	C-N-	Oxi	Osmo
SD		*uvrA*						*entF*		
CS				*groL* *nusA* *aceE* *gadC*	*uvrA*			*uvrA*	*uvrA* *cydA*	*dnaX* *uvrA* *kat*
HS									*livJ*	
pH						*cydA*	*cydA*	*cydA*		*mreBCD*
UV										
C-										
N-										
C-N-										
Oxi										
Osmo

**Table 7:** Differentially expressed stress genes in each pair of conditions for C58

	SD	CS	HS	pH	Fe-	Oxi	CS and Oxi	pH and CS	pH and HS	pH and Fe-
SD										
CS				*sit*						
HS				*groEL* *vir*						*sit*
pH						*groEL*		*dnaK*	*groEL* *dnaK* *vir*	
Fe-						*sit*			*sit*	
Oxi										*sit*
CS and Oxi										
pH and CS										
pH and HS										*sit*
pH and Fe-										

It is worth noting that SAM generally detected more differentially expressed stress response genes than ANOVA can confirm. For example, the heat shock response gene *hslO* was detected by SAM even though ANVOA was not able to confirm that its means are significantly different among the treatment conditions for *E. coli* MG1655. More interestingly, many stressful conditions triggered *uvrA* gene expressions, which is the SOS response gene in *E. coli* MG1655. For *A. tumefaciens* C58, stress response genes are induced more by combined treatment conditions. For example, many stress response genes are differentially expressed not just by heat shock but by heat shock and low pH combined. We can conclude that the tiling microarrays detected sensible gene expression changes that conform to our expectation with regard to known cell stress response gene behaviors in the two tested bacteria.

#### Novel transcript discoveries

One of the stated benefits of a tiling microarray is that it can detect unexpected expressions as well as annotated gene expressions. Indeed, the two tiling microarrays for *E. coli* MG1655 and *A. tumefaciens* C58 detected significant numbers of RNA expressions from non-gene-coding regions on the two genomes. For example, our tiling microarray detected all 65 non-coding regulatory RNAs (ncRNAs) annotated in GenBank report U00096.3 for *E. coli* MG1655. Among the 65 ncRNAs, *dicF* is computationally predicted to target the *hslV* gene according to the bacterial small regulatory RNA database (BSRD) [47]. Since our tiling microarray covers both the *dicF* ncRNA and its target gene, we can calculate the correlation value between them is −0.4819401, which agrees with the predictions. Because the whole genome is monitored, when there are new predictions of such regulatory activities, the correlation values can be extracted from our tiling microarray data without having to design new experiments to validate the predictions. We have seen many such evidences of ncRNA and target gene correlations, but it will require more analysis and maybe some independent validation experiments to report their biological functions. It suffices to say here that the two tiling microarrays do allow novel transcript discoveries as we have anticipated.

## Conclusions

In this work we have described the design strategies and validation experiments of two whole-genome tiling microarrays for *E. coli* MG1655 and *A. tumefaciens* C58 bacteria. The tiling microarrays are thermodynamically optimal for the two genomes based on the rigorous calculations conducted by the PICKY software [17]. This means that all probes selected have maximum specificity toward their target genome regions and no additional probes can be added to the microarrays without jeopardizing its specificity under the given design constraints. In average, there is a unique microarray probe every 100 bp along the genomes to uniquely detect any transcripts coming from that region. Therefore, transcripts longer than 100 bp are likely to be detected by at least one tiling microarray probe. The 100 bp selection window can be adjusted upward or downward depending on the microarray probe count and user preferences, but we do not expect the selected probes to increase significantly when this window is reduced because most thermodynamically optimal probes, if not all, should have been found by PICKY at the current 100 bp window size given the ~40 bp non-overlapping probe length.

The tiling microarray probes can detect transcripts expressed from both strands of the genomes because most of the common cDNA conversion and labeling protocols automatically produced double-stranded DNAs from original RNA transcripts. Given gene annotation information and bioinformatic techniques such as gene predictions, determining the actual strand of expression is not difficult for most genes [48]. One can also use some other methods such as RT-PCR to confirm the expressing strand for a few difficult transcripts.

We believe tiling microarrays are useful for many gene expression studies, especially for novel and non-model species that have not been annotated yet. Actually, tiling microarrays can help identify novel gene expressions and facilitate the annotation of novel species. Microarrays tend to produce data much faster (in just 2 days), can tolerate a few mismatched bases due to polymorphisms or sequencing errors, tend not to be overwhelmed by excessive bacterial rRNAs as RNA-Seq does, and does not usually require sophisticated computing capacity to interpret the data. The software used in this study and the data produced by the experiments are freely available to other researchers who may wish to design their tiling microarrays.

## Additional files

10.1186/s13104-016-2113-4 Picky analysis results for a previously designed tiling microarray. The tiling microarray created by Yu, et al. [21] was designed using a pipeline [22] that depends on BLAST for sequence-level probe specificity screening. This compressed ZIP archive contains the summary (W83.report) and detail analysis (W83.picky) produced by Picky for this microarray.
10.1186/s13104-016-2113-4 Assembled genome sequences for *E. coli* MG1655 and *Agrobacterium tumefaciens* C58. Two FASTA files containing the assembled genomic sequences are provided in this compressed ZIP archive.

## Abbreviations

NGS: next-generation sequencing
*E. coli*: *Escherichia coli*
*A. tumefaciens*: *Agrobacterium tumefaciens*
GEO: gene expression omnibus
RMA: robust multi-array analysis
CCC: concordance correlation coefficient
SAM: significant analysis of microarray
BSRD: bacterial small regulatory RNA database
